# Supplementary figures and images for: Early Feeding Regime of Waste Milk, Milk, and Milk Replacer for Calves Has Different Effects on Rumen Fermentation and the Bacterial Community
Source: Animals (Basel). 2019 Jul 15;9(7):443. doi: 10.3390/ani9070443 (PMC6680522; doi:10.3390/ani9070443)

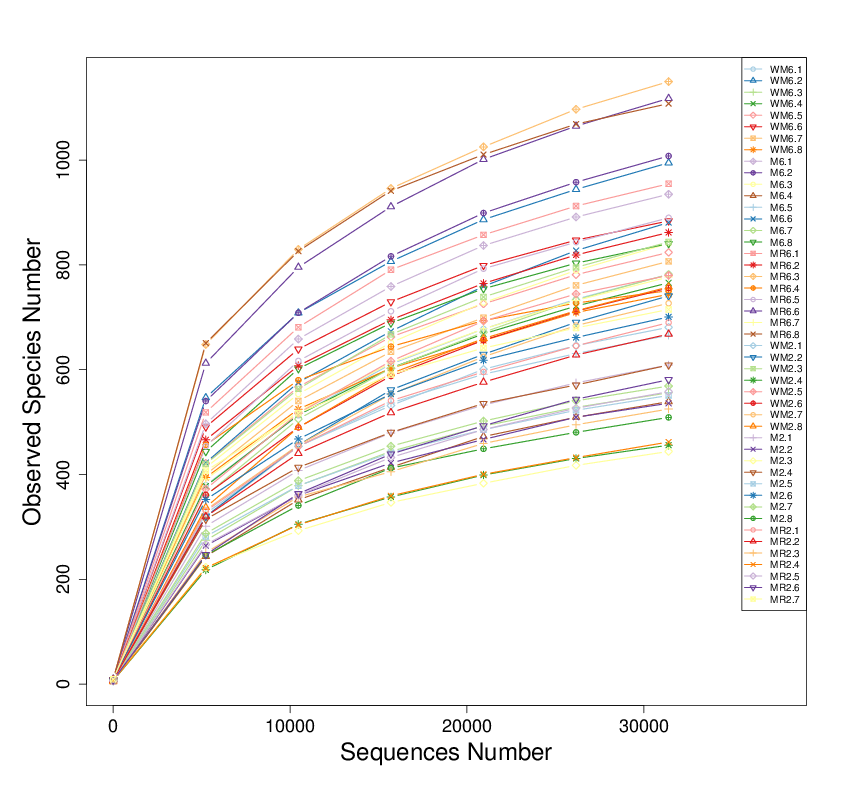

Supplement: Supplementary file 1 [file animals-09-00443-s001.zip › supplement revised/fig S1.png]
